# In silico clinical trials for relapsing-remitting multiple sclerosis with MS TreatSim

**DOI:** 10.1186/s12911-022-02034-x

**Published:** 2022-11-15

**Authors:** Fianne L. P. Sips, Francesco Pappalardo, Giulia Russo, Roberta Bursi

**Affiliations:** 1 InSilicoTrials Technologies, ’s-Hertogenbosch, Netherlands; 2 Department of Drug and Health Sciences, University of Catania, Catania, Italy; 3 Mimesis Srl, Catania, Italy

**Keywords:** Relapsing-Remitting Multiple Sclerosis, In silico trials, Digital patient, Computational modeling and simulation, Study design

## Abstract

**Background:**

The last few decades have seen the approval of many new treatment options for Relapsing-Remitting Multiple Sclerosis (RRMS), as well as advances in diagnostic methodology and criteria. These developments have greatly improved the available treatment options for today’s Relapsing-Remitting Multiple Sclerosis patients. This increased availability of disease modifying treatments, however, has implications for clinical trial design in this therapeutic area. The availability of better diagnostics and more treatment options have not only contributed to progressively decreasing relapse rates in clinical trial populations but have also resulted in the evolution of control arms, as it is often no longer sufficient to show improvement from placebo. As a result, not only have clinical trials become longer and more expensive but comparing the results to those of “historical” trials has also become more difficult.

**Methods:**

In order to aid design of clinical trials in RRMS, we have developed a simulator called MS TreatSim which can simulate the response of customizable, heterogeneous groups of patients to four common Relapsing-Remitting Multiple Sclerosis treatment options. MS TreatSim combines a mechanistic, agent-based model of the immune-based etiology of RRMS with a simulation framework for the generation and virtual trial simulation of populations of digital patients.

**Results:**

In this study, the product was first applied to generate diverse populations of digital patients. Then we applied it to reproduce a phase III trial of natalizumab as published 15 years ago as a use case. Within the limitations of synthetic data availability, the results showed the potential of applying MS TreatSim to recreate the relapse rates of this historical trial of natalizumab.

**Conclusions:**

MS TreatSim’s synergistic combination of a mechanistic model with a clinical trial simulation framework is a tool that may advance model-based clinical trial design.

## Background

In the past decade, the landscape of Relapsing-Remitting Multiple Sclerosis (RRMS) treatment has been transformed. The number of approved disease modifying therapies (DMTs) for MS has increased quickly, with currently over 10 DMTs on the market [1, 2]. The wide selection of first- and second-line therapies available for RRMS means there are multiple treatment options available for patients with both mild and moderate disease. Simultaneously, earlier and more sensitive diagnosis of RRMS has been facilitated by improvements in diagnostic criteria and methodology [3].

These developments have led to increased options and a better outlook for patients. However, they have also complicated not only treatment planning for RRMS, but also designing and executing successful clinical trials for new DMTs [4]. Firstly, with so many efficacious treatment options available, new DMTs are less often tested against placebo, but rather need to show a benefit with respect to existing DMTs. Second, the availability of more sensitive diagnosis and more effective treatment have resulted in a change in the typical clinical trial population—current trial populations generally consist of patients with lower annual relapse rates and less advanced disease than populations in historical trials [4].

These changes influence trial design in several different ways. A milder disease with fewer relapses results in the necessity for longer, larger, and thus more expensive clinical trials. Additionally, determining the optimal trial design is complicated by the fact that extrapolating the effect of the existing medication currently used in control arms from “historical” patients’ populations to patients’ populations included in today’s trials can be a challenge.

To support clinical trial design optimization and therefore to increase drug development programs success chances we have developed MS TreatSim, a web-based product which leverages a mechanistic, agent-based model of the immune system and its dysregulation in RRMS [5, 6]. The product includes mechanisms of action (MoA) and quantitative effects for four commonly used RRMS treatments (IFNβ-1a, teriflunomide, natalizumab and ocrelizumab) at various doses and regimens. Here, we show that the simulator can be used to select heterogeneous patients’ populations with a tailored level of disease severity, allowing the user to switch between mild and moderate disease stages and to investigate the effects of existing drugs. Finally, in this use case, we illustrate how the simulator can be used to reproduce a historical trial by simulating and reproducing the relapse rates of the AFFIRM phase III trial on natalizumab [7].

## Methods

MS TreatSim (InSilicoTrials Technologies SpA, *mstreat.insiliconeuro.com*) is a web-based product available on the cloud-based InSilicoTrials.com platform [8]. The platform exploits Microsoft Azure infrastructure to allow large-scale simulations with real world computation times of minutes to hours [9]. MS TreatSim is available through a Software as a Service (SaaS) delivery model, with pay-per-use pricing (additional information on tool access can be found at https://insilicotrials.com/mstreatsim).

MS TreatSim creates groups of digital patients (DPs), to which customizable simulation workflows reflecting selected treatment plans are applied. MS TreatSim leverages a mechanistic model of the immune system, the auto-immune response that characterizes RRMS, and four commonly prescribed treatment options.

### The simulation framework and the mechanistic model underlying MS TreatSim

The model was built in the Universal Immune System Simulator (UISS) framework [10]. UISS incorporates a detailed model of the innate and adaptive immune system, implemented using the agent-based modelling paradigm. The framework has been employed to simulate and support a variety of pathogen responses, vaccine mechanisms and immune disorders, including tuberculosis vaccination [11, 12], citrus-derived vaccine adjuvants [13], and COVID-19 infection and vaccination [14, 15], and has undergone various validation and verification procedures [16]. The framework is flexible and multifunctional, allowing expansion of the basic immune functionality to specific disease pathology and treatment MoA [10].

In UISS, cells of the immune system are modelled as agents—entities that are followed in an individual way. Such an implementation allows a realistic representation of the complex interactions and stochasticity of the human immune system. The model incorporates the interactions and behaviors of the main lymphocytes of the immune system, including B cells, T helper cells, T regulatory cells, cytotoxic T cells, and natural killer cells. The flexible agent-based core is modelled on a spatial grid and combined with a binary string-based implementation of receptor ligand binding, hematopoiesis and thymus selection, and cytokine signaling.

For application to RRMS, the model was extended with the basic components and tissues of multiple sclerosis [5]. Spatially, the RRMS model includes a white matter compartment, populated with oligodendrocytes. These oligodendrocytes may be attacked by the immune cells via the auto-immune response to the oligodendrocyte-associated self-antigen. In the simulation, the recognition of such self-antigens is triggered by an event replicating an Epstein-Barr Virus infection [5, 17]. Reductions in oligodendrocyte numbers as a result of auto-immune events are interpreted as relapses. Immune heterogeneity and the main characteristics determining disease severity (e.g. age of onset, lesion load, oligoclonal band status) are incorporated via calibrated model settings and parameters, so that the final model provides an individualized, tailored, simulation of RRMS. Further details are reported in [5, 11, 12, 14, 16, 18].

Finally, treatment for RRMS is implemented by incorporating the mechanism of four commonly prescribed DMTs (IFN-β1a, teriflunomide, natalizumab or ocrelizumab) at the (sub-)cellular level. Natalizumab, for example, is implemented via its net effect on leukocyte migration in the white matter compartment [5]. Since treatments are incorporated via their mechanisms, the personalized effects of treatment simulated with the model are expressed not only in terms of high-level statistics like the relapse rate, but also on the underlying immune dynamics. Additional model details can be found in [5].

**Fig. 1:**
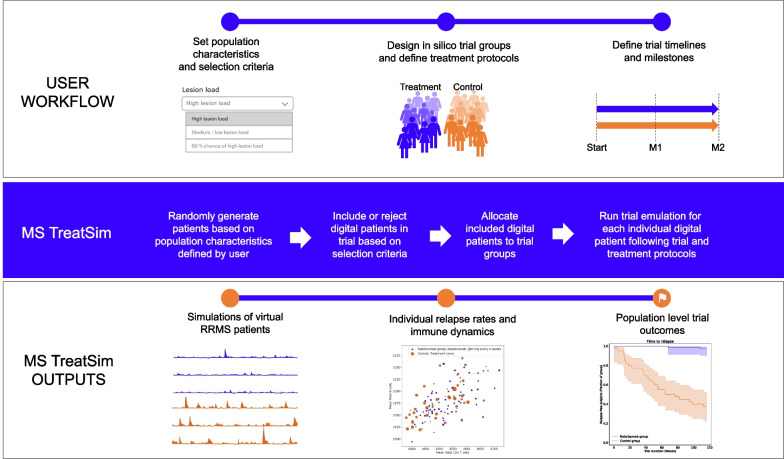
MS TreatSim workflow. In the first step, the user sets up the simulation by selecting population characteristics and inclusion/exclusion selection criteria (see also Fig. 2). Next, the user defines the trial groups and treatment strategies to be simulated and compared. Finally, the user defines the trial timelines including its duration and any additional intermediate analysis milestones of the trial. MS TreatSim’s simulation framework next generates DPs based on the defined population and includes them into the digital patient groups. Finally, each DP is simulated individually according to the group’s treatment of choice. The outputs generated by MS TreatSim are built on the individual simulations of each DP and are displayed in terms of clinical endpoints descriptive statistics, individual profiles, bubble and Kaplan–Meier plots

MS TreatSim, combines the UISS-based model with a simulation framework for in silico trial simulation (Fig. 1). The simulation framework converts user inputs (top row Fig. 1) to in silico trial outputs (bottom row Fig. 1) through several steps. In MS TreatSim, each DP taking part in an in silico trial consists of a personalized model instance. To set up a trial, several user inputs are required: the characteristics defining the population of DPs, the inclusion and exclusion population selection criteria, the groups, group sizes and treatments to be simulated in the trial, and finally the trial’s timelines and additional interim clinical endpoints analyses during the trial.

**Fig. 2:**
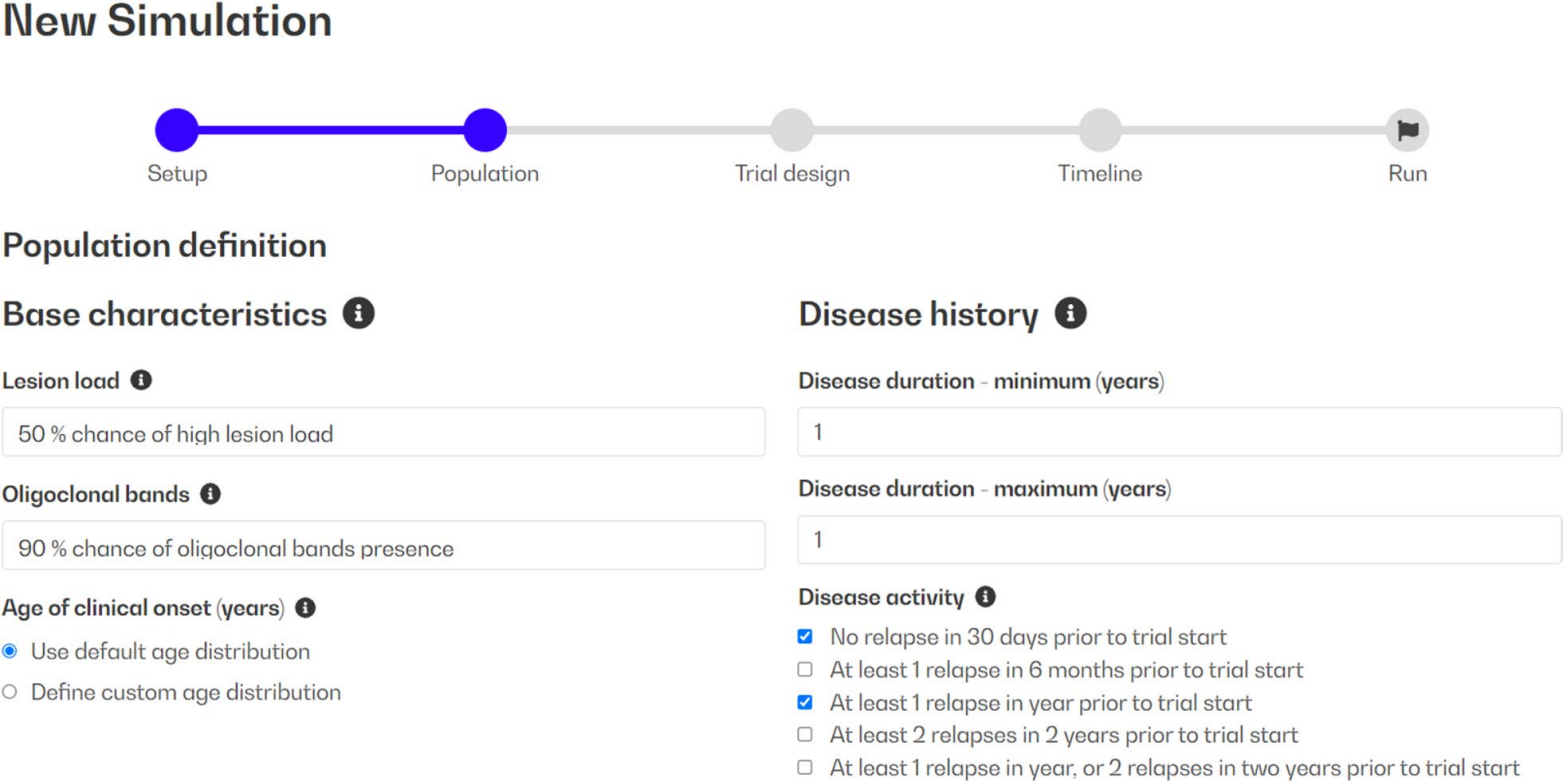
Digital patients’ population set-up: first step in MS TreatSim workflow. The user selects the distributions of base characteristics in the population (high vs low/medium lesion load, oligoclonal bands presence, age of onset, and disease duration in the population). In the panel disease activity, the user selects the criteria by which the DPs will be included or excluded

The patients’ population base characteristics the user may define (Fig. 2) include lesion load (user may select high, low/medium, or 50% chance of high), oligoclonal band status (user may select present, not present, or 90% chance of presence) and age of onset (distributed over the categories of 18–29/30–39/40–49 years). These characteristics have been mapped to model parameters through a calibration and validation process [5], and so can be used to set up individualized models. Further model settings include immune system initialization parameters and disease duration. Patients’ inclusion/exclusion selection criteria, finally, mirror disease activity criteria that represent common selection criteria for RRMS clinical trials.

To simulate the trial, the user-defined patients’ population base characteristics are firstly translated into a simple uniformly distributed statistical model of base characteristics distributions. This statistical model is used to create heterogeneous individual models with the aid of random sampling. Next, the inclusion and exclusion criteria are applied for DP inclusion. The included DPs are then randomised into the pre-defined groups and prepared for simulation. Finally, each DP is simulated according to the protocol defined by the treatment options and timelines.

As a last step, the individual simulations in each group are analyzed and used to generate high-level statistics on clinical endpoints for the different patients’ groups. In addition, the underlying detailed and time continuous DPs allow the user to zoom in to individual details or immune variables.

### Application of MS TreatSim to create heterogeneous digital patients’ populations

To demonstrate how changing MS TreatSim selection criteria leads to digital patients’ populations that behave differently, we chose and set up two distinct populations (Populations 1 and 2). For the creation of Population 1, we selected DPs with an age of disease onset in the categories 30–39 and 40–49 years and high lesion load, whereas Population 2 was generated with age of onset in the youngest category (18–29 years) and a low/medium lesion load. All DPs in Populations 1 and 2 were simulated for 5 years, and only DPs that had developed relapses (and thus RRMS symptoms) in that period were selected. Finally, 200 DPs were included in Population 1 (58% of simulations had at least 1 relapse), and 200 in Population 2 (59% of simulations had at least 1 relapse).

### Historical trial for natalizumab

Results of the AFFIRM clinical trial for natalizumab [7] were first published 15 years ago. In this phase III trial, the treatment group consisted of 627 patients who received 300 mg of natalizumab every 4 weeks. The control group was a placebo group, consisting of 315 patients. After 104 weeks of treatment, 67% of the treatment group remained relapse free, compared to 41% of the control group. This was accompanied by a 68% reduction in annual relapse rate, an 83% reduction in T2 weighted lesions determined by MRI, and a 92% reduction in gadolinium-enhanced lesions. Due to lack of raw trial data availability, the recreation focused on recreating the design and the global characteristics of the trial as published in [7].

### Application of MS TreatSim to recreate a historical trial

As a case study to illustrate the application of the product, the AFFIRM clinical trial for natalizumab was reproduced with MS TreatSim. Two groups of DPs that mirror the historical population were created with a treatment control ratio of 2:1, mirroring the AFFIRM trial [7]. The inclusion criteria and base characteristics of the in silico trial were chosen to reproduce the base characteristics in the AFFIRM trial [7]; the age of onset distribution was set to 49.4%: 36.2%: 14.4% (18–29, 30–39 and 40–49 years), lesion load was set to *high* and oligoclonal bands status to *present* for all DPs, simulation duration prior to the trial was set at 5 years, and DPs were only included if they had experienced at least one relapse in the year preceding inclusion, but not in the final month. The natalizumab group (n = 80) was treated in silico with 300 mg of natalizumab every 4 weeks for 104 weeks, while the control group (n = 40) remained treatment naïve.

### Statistical analysis

Statistical analysis of the digital patients from Populations 1 and 2 was performed in R. Tests were performed to determine whether the immune variables were from the same distribution using the two sample Kolmogorov–Smirnov test [19] and to compare the distributions medians using a two-sided Wilcoxon Rank Sum test [20]. The 95% confidence intervals for the Kaplan–Meier estimators were calculated by means of Greenwood’s formula [21], and plotted with the aid of the lifelines [22] package in python. In the in silico trial, all trial durations were assumed to be maximal and patients were not censored.

## Results

In this section, the results obtained for the two applications described above—the generation of heterogeneous digital patients’ populations, and the recreation of the main characteristics of a historical trial, will be presented and discussed.

### Application of MS TreatSim to create heterogeneous digital patients’ populations

**Fig. 3:**
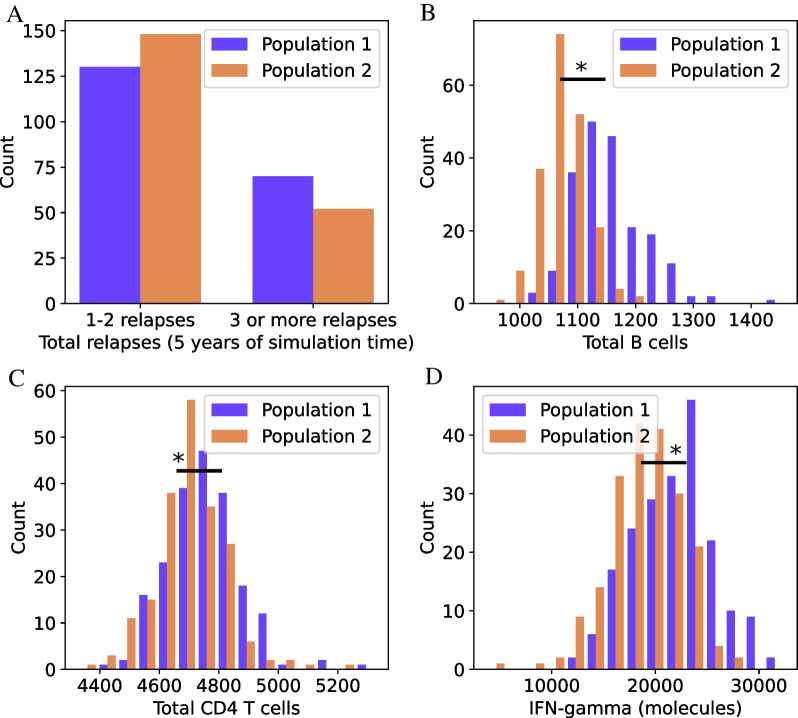
Distribution of disease and immune system characteristics for Populations 1 and 2. Each population consists of 200 DPs. **A** Histogram showing the total number of relapses over 5 years, divided between 1 or 2 relapses, or three or more. **B** Mean total B cells over 5 years. Distributions and medians of the mean B cell number were both found to be significantly different between Populations 1 and 2 (*p* < 0.001). **C** Mean total CD4+ T cells over 5 years. Distributions and medians of the mean CD4 + T cells were both found to be significantly different between Populations 1 and 2 (*p* < 0.001). **D** Mean IFN-γ over 5 years. Distributions and medians of the mean IFN-γ concentration were both found to be significantly different between Populations 1 and 2 (*p* < 0.001). * = distributions and medians difference, *p* < 0.001

To test how heterogeneous populations generated by MS TreatSim can be, two distinct populations—Populations 1 (older age of onset categories, high lesion load) and 2 (youngest age of onset category, low/medium lesion load) were generated. Each population consisted of 200 DPs. In Fig. 3, the resulting relapse rates (Fig. 3A) and immune system dynamics (Fig. 3B–D) including the statistical results are displayed.

The results show a clear shift in relapse rates and statistically significant differences (*p* < 0.001) in distributions and medians for the immune variables as a result of the different base characteristics—mirroring two distinct subpopulations of RRMS patients with differences in (mean) disease activity. Moreover, the results also clearly show that the model allows for a large patient’s heterogeneity.

### Application of MS TreatSim to recreate a historical trial

**Fig. 4:**
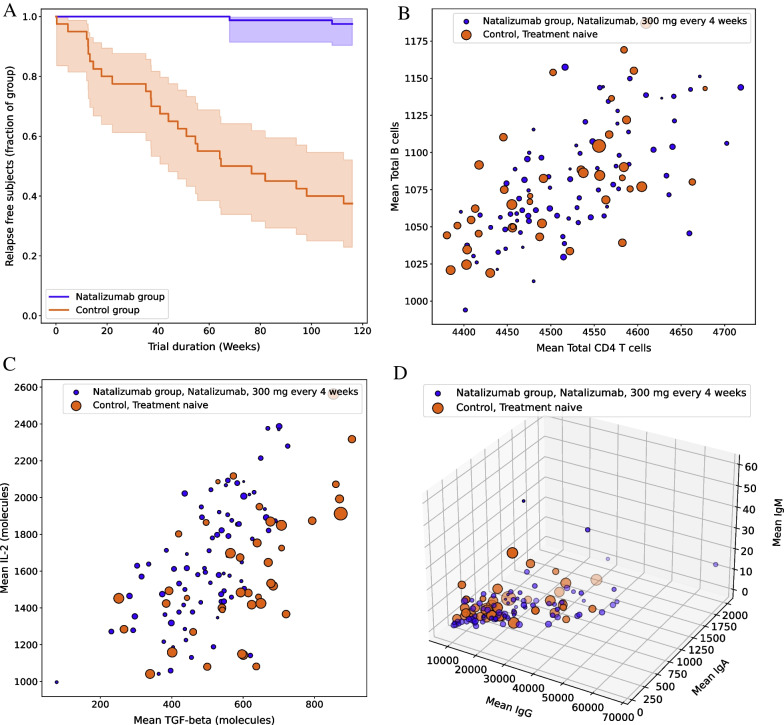
In silico trial of natalizumab. **A** Kaplan–Meier plot shows simulated patients’ survival to first relapse including 95% confidence intervals for the natalizumab and placebo treatment groups. Note that the duration mirrors AFFIRM maximal time span of 116 weeks, whereas most analyses were performed at 104 weeks. **B** Individual leukocyte concentrations. Means (over length of trial, per DP) of the total number of B cells versus total number of CD4 T cells. **C** Individual cytokine concentrations. Means (over length of trial, per DP) of the IL-2 molecules present in the DP versus TGF-β. **D** Individual antibody concentrations. Means (over length of trial, per DP) of the IgA, IgG and IgM molecules. Blue = natalizumab group; Orange = control (treatment naive) group. In **B**–**D**, all points are scaled by the individual’s relapse activity, so that a larger dot indicates a DP with more cumulative loss of oligodendrocyte and thus a higher relapse rate

As a test case for the recreation of a historical trial, the AFFIRM clinical trial for natalizumab was recreated [7]. In the phase III trial AFFIRM, the dose was 300 mg administered every 4 weeks. The trial was simulated by means of the built-in MoA for natalizumab, based on reduction in leukocyte migration (see Methods), and results are shown in Fig. 4A. 80 DPs were simulated for the treatment group and 40 DPs for the control group. In the historical trial [7], the effect of treatment on relapse rate was large. After two years, 67% of patients remained relapse free in the treatment group, versus only 41% in the placebo group. The improvement was even more pronounced in the emergence of new or enlarging lesions; there was an 83% reduction in the occurrence of new or enlarging hyperintense (determined by T2-weighted magnetic resonance imaging) lesions, and a 92% reduction in lesions determined by gadolinium-enhanced magnetic resonance imaging.

In the in silico trial, at 104 weeks the percentage of relapse free subjects was larger than 90% in the natalizumab group, whereas for DPs in the control group this percentage dropped to 40%. These results show that the simulated relapse rates successfully replicated the observed relapse rates in the control group since the value of 41% observed in the historical trial is well contained within the estimated 95% confidence intervals of the simulated relapse rates. The simulated natalizumab treatment effect, on the other hand, resulted in over 90% relapse free patients at 104 weeks trial overestimating the 67% relapse free observed in the historical trial and appearing more in line with the T2 weighted and gadolinium-enhanced lesions reductions data observed in that trial. Finally, the effect of treatment on cytokine and leukocyte levels was quantified. The natalizumab treatment can be seen to affect cytokine levels (Fig. 4C), while the overall T and B cell numbers (Fig. 4B) and antibody concentrations (Fig. 4D) are similar between treatment and control groups.

## Discussion

Advances in the diagnosis and available disease modifying therapies for RRMS have improved patient care and expanded treatment options for RRMS patients. However, increasing treatments options also complicates treatment planning and drug development. Simulation models can be valuable tools to support clinicians and researchers, by giving insight into the relationships between (sub)population, immune dynamics, and treatment effects. This study has shown that MS TreatSim can be used to create heterogeneous populations of DPs with variable immune system responses and can mirror qualitatively response to treatment in a RRMS population in a use case of a historical natalizumab trial.

First, we illustrated how heterogeneous, differently behaving populations of RRMS patients can be created by setting up different populations with the aid of the integrated base characteristics. The results showed that choosing a different subpopulation in MS TreatSim indeed resulted in the corresponding shift of the mean disease activity. In addition, they also clearly showed that the heterogeneity of the population—which is a hallmark of the true RRMS population [23]—is mirrored in the in silico population. For example, the range of the number of relapses an individual experienced over 5 years spans from 1 to 9 relapses. Although the relationship between base characteristics such as age and MS progression is complex, the use of age and lesion activity leads to a realistic effect on relapse activity.

Secondly, we used such populations as a starting point to recreate in silico the setup of the AFFIRM trial for natalizumab. We performed this simulation with a modest sample size, which is adequate in this case as the treatment effect is large and so the results are robust. In the control group, the percentage of relapse free subjects in the in silico trial reproduced the percentage of relapse-free subjects in the AFFIRM trial well. In the simulated treatment group, the treatment effect was clearly visible, although the percentage of relapse free subjects was higher in the simulation than in the trial. For a correct interpretation of these results, several characteristics of the study and of the treatment of RRMS must be considered. While the percentage of relapse free subjects generally is used as a good high-level measure of disease activity in a group, a comprehensive evaluation of the effect of treatment on inflammatory outcomes includes also measures like the reduction in the annualized relapse rate (ARR), and the MRI-determined lesion load. These measures were all improved to a greater extent than the percentage of relapse free subjects in the AFFIRM trial. Therefore, if the in silico natalizumab treatment effect is also compared with the improvement seen in lesion load reduction, which is a good metric for disease activity, and also closely relates to the simulated relapse rate, one can conclude that the model adequately predicts the clinical improvement observed in natalizumab treated patients (Fig. 3A). Additionally, in real-world studies that were performed after market authorization of natalizumab, the reduction in ARR was larger than in the AFFIRM trial [23, 24]. The AFFIRM trial found the relapse rate to be reduced by 68% after 1 year, whereas the 22 observational studies analyzed in [23] found ARR to decrease by 73–94% under natalizumab treatment, and the 10 year real world study reported in [24] found the ARR to be reduced by 92.5% across the population. An explanation for this may lie in the observation by real-world studies that disease history may affect the patient’s potential to respond to natalizumab—for example, patients with a more complex treatment history and a higher baseline Expanded Disability Status Scale (EDSS) are less likely to see a positive effect from natalizumab treatment [24]. Since, in this use case, the disease and treatment history of the patients in the AFFIRM study were described in a generic manner, patients with complex disease histories may be underrepresented, and this factor may also play a role in the overestimation of the percentage of relapse free subjects in the treatment group.

Another constraint in this study might well be attributed to the type of data, i.e., descriptive and synthetic data instead of raw data, that was available for the recreation of the historical trial and that therefore limited a detailed analysis of the simulated and historical trial results. If, on one hand, lack of such analysis limits the extent of corroboration of our conclusions, on the other hand, the presented results simultaneously demonstrate the broad utility of MS TreatSim. Using only high-level data, a completely independent reproduction of the test trial could be created, and meaningful and useful results could be generated. Although the results presented here do not represent a complete and systematic statistical evaluation of MS TreatSim’s performance against the available literature—for example, only one of the four included treatment options has been simulated—the test case clearly demonstrates the potential utility of MS TreatSim at the clinical trial level. Future work will aim to further develop MS TreatSim and its underlying model and integrate and refine included treatment strategies to improve the precision of the individualized predictions, and to more comprehensively compare model performance against historical clinical trials and available real world data.

MS TreatSim leverages a detailed, (sub-)cellular mechanistic model of RRMS for a population-level application. A number of alternative modelling approaches simulating the population level disease activity and progression in RRMS have been suggested in recent years [25–31] reflecting the high level of interest in model-based decision and design support. Many of these modelling approaches are based on statistical or artificial intelligence methodology, and thus necessarily are fully data-driven, and focus on clinical markers of RRMS. The agent-based approach of the model underlying MS TreatSim is qualitatively different and complementary—instead of focusing on prognosis of disease progression (expressed as EDSS) or disease activity (expressed as e.g., ARR) based on data alone, the mechanistic model builds on knowledge of the immune system, knowledge of the disease and specifics of the MoA of the treatments, in addition to data. In doing so, it provides an opportunity to investigate not only effects on clinical outcomes, but simultaneously details the underlying immune dynamics. In MS TreatSim the treatment effect can be examined at every level, from high level outcomes to individual responses, to the most detailed immune dynamics.

MS TreatSim has been developed and validated [5] in collaboration with neurologists specialized in RRMS, and currently finds numerous applications within the pharmaceutical clinical development of new therapeutics targeting RRMS. These applications consist of supporting clinical trial design processes including definition of group sizes, timelines, and patients’ subpopulations. Additionally, MS TreatSim is used to predict real-world setting relapse rates and create synthetic arms, it provides support for clinical decision making, and it aids design of novel treatment strategies. Finally, the intrinsic flexibility of MS TreatSim also offers opportunities for expansion of the simulation workflow and/or underlying model towards a wider array of applications. For example, the simulations can be expanded to investigate combination therapy, to incorporate additional mechanisms for new and existing drugs, and to map personal or clinical characteristics even more closely to model parameters.

## Conclusion

This study has shown that MS TreatSim can be used to create heterogeneous populations of DPs that have large variations in immune system responses and thus can display different responses to treatment, mirroring the variability of the real-world population of RRMS patients. The test case of recreating an historical trial of natalizumab generated a treated population that displayed a clear relapse rate reduction with respect to placebo, as observed in the clinical trial. Even though the wide range of responses observed in the literature to natalizumab and other treatment options and their relationship to disease history and historical context will require further exploration in the future, the current case study already illustrates how the mechanistic model and the simulation framework synergistically combined in MS TreatSim can be used to inform clinical trial design. Moreover, MS TreatSim can be envisaged also as a valid support in assisting the neurologist in the choice of the best treatment regimen accordingly to the patient immunological and disease progression profile.

## Data Availability

The data that support the findings of this study are available from InSilicoTrials Technologies, but restrictions apply to the availability of these data, which were used under license for the current study, and so are not publicly available. Data are however available from the authors upon reasonable request and with permission of InSilicoTrials Technologies. For inquiries, please contact Roberta Bursi (roberta.bursi@insilicotrials.com).

## Abbreviations

ARR: Annualized relapse rate
DMT: Disease modifying therapy
DP: Digital patient
EDSS: Expanded disability status scale
MoA: Mechanism of action
RRMS: Relapsing-Remitting Multiple Sclerosis
